# Real-World Clinical Impact of Using Personal Glucose Targets in a Hybrid Closed-Loop System Differs According to Age

**DOI:** 10.1177/15209156251376010

**Published:** 2025-09-05

**Authors:** Julia Ware, Simon Bergford, Peter Calhoun, Judy Sibayan, Malgorzata E. Wilinska, Yue Ruan, Roman Hovorka

**Affiliations:** 1 Institute of Metabolic Science-Metabolic Research Laboratories, University of Cambridge, Cambridge, United Kingdom.; 2 Department of Paediatrics, University of Cambridge, Cambridge, United Kingdom.; 3 Jaeb Center for Health Research, Tampa, Florida, USA.

**Keywords:** artificial pancreas, automated insulin delivery, closed-loop, glucose target, personalized medicine, type 1 diabetes

## Abstract

**Objective:**

CamAPS FX is a customizable hybrid closed-loop app with a default target glucose of 105 mg/dL. The personal glucose target is user-adjustable in 1 mg/dL increments between 80 and 198 mg/dL in 30-min segments over 24 h. We assessed the impact of different personal glucose targets on glycemic control during real-world use of CamAPS FX in different age-groups.

**Methods:**

We retrospectively analyzed data from real-world CamAPS FX users from 11 countries across all age-groups (1 to 90 years), who used the system between December 1, 2022 and November 30, 2023, and had a minimum of 8 weeks of closed-loop use. Every sensor glucose reading was matched to the user-specified glucose target.

**Results:**

In total, 8604 users (mean age 32 ± 19 years, median days of data 89 [IQR 59, 119]) were included. Personal glucose targets were most frequently used by very young children (>50%), followed by school-aged children (>40%). All other age-groups used the default target 65%–68% of the time. Overall, personal glucose targets >120 mg/dL were associated with time in target range <70%. Time <70 mg/dL remained <4% across targets, apart from at the lowest (80–89 mg/dL). Older adults achieved time in range ≥70% across all targets. Very young children and young adults were only able to achieve time in range >70% with targets set below the default, which was associated with time <70 mg/dL of >4% in very young children.

**Conclusions:**

Personal glucose targets are frequently used, with clinical impact differing depending on user-age. Adjusting glucose targets may help to achieve recommended glycemic targets and individual glycemic goals.

## Introduction

A multitude of randomized controlled trials have shown that hybrid closed-loop insulin delivery improves glycemic outcomes and quality of life across all age-groups.^1–5^ Real-world evidence shows consistent results with clinical studies, and use of hybrid closed-loop systems as treatment for type 1 diabetes is now becoming standard of care.^6^

More recently, studies have started to explore the clinical impact of user-customizable features of these systems, with one particular area of interest being customized glucose targets. Several commercially available hybrid closed-loop systems include options for setting different glucose targets (iLet Bionic Pancreas, 780G SmartGuard, Omnipod 5, Diabeloop and CamAPS FX), so that users are able to customize the algorithm to their individual needs. Analysis of real-world data from some of these systems has shown that different glucose target settings are associated with differences in glycemic control, namely lower target settings being associated with higher time in target range.^7,8^

CamAPS FX is an inter-operable, customizable, adaptive hybrid closed-loop app with a default glucose target of 105 mg/dL. The personal glucose target is user-adjustable in 1 mg/dL increments between 80 and 198 mg/dL in 30-min segments over 24 h. Other systems offer a maximum of five different glucose target settings, and only one other system allows for more than two target settings in a 24-h period (Omnipod 5 with a maximum of eight).^9^ We previously evaluated the effect of different glucose targets in a post-hoc analysis using data from five randomized controlled trials, which showed that discrete study populations had differences in glycemic control when applying similar glucose targets.^10^ However, findings were limited by a relatively small cohort of participants exclusively from clinical trials. In the present retrospective real-world analysis of a large cohort, we aimed to explore the clinical impact of a wide range of personal glucose targets available within the CamAPS FX app, including potential differences between age-groups.

## Methods

### Design

We retrospectively analyzed data collected from users of the CamAPS FX hybrid closed-loop app (CamDiab Ltd, UK) between December 1, 2022 and November 30, 2023 from 11 countries (Germany, United Kingdom, Austria, Netherlands, Australia, Switzerland, Poland, Czechia, Sweden, Spain, and Italy). Users were included in the analysis if they consented for their data to be analyzed and used closed-loop for a minimum of 8 weeks.

### Closed-loop system

The CamAPS FX smartphone app (Android operating system at the time of data extraction) is integrated with the mylife YpsoPump insulin pump (Ypsomed AG, Switzerland) and has the following sensor options, Dexcom G6 glucose sensor (Dexcom, CA, USA) or the FreeStyle Libre 3 and 3 Plus (Abbott, IL, USA) (Supplementary Fig. S1). Only those using Dexcom with CamAPS FX were included in the present analysis, due to Freestyle Libre 3 rollout not commencing until after the study start date. The app-based model-predictive control algorithm incorporating adaptive learning automatically adjusts insulin delivery every 8 to 12 min to achieve a default nominal glucose target of 105 mg/dL. CamAPS FX offers personalizable, user-initiated modes of operation, including user-selectable glucose targets between 80 and 198 mg/dL. The personal glucose target can be adjusted in 1 mg/dL increments across different times of day, the shortest selectable time segment being 30 min.

### Data analysis and statistical methods

For each user, every sensor glucose reading was matched to the user-specified glucose target. Glucose endpoints were calculated for each target if the user had ≥4 h in the respective target. Only data from periods when closed-loop mode was operational were included. All endpoints were calculated per user, giving equal weight to each user.

Analyses were conducted across all users, and across six pre-defined age groups: 1–6 years, 7–13 years, 14–17 years, 18–22 years, 23–65 years and ≥66 years old. Analyses were conducted overall, during daytime (06:00 to 23:59), and during nighttime (00:00 to 05:59). Glucose targets were collated into target ranges for the analysis: 80–89 mg/dL, 90–99 mg/dL, 100–119 mg/dL, 120–139 mg/dL, 140–159 mg/dL, 160–179 mg/dL, and 180–199 mg/dL. Mean sensor glucose, time spent in range 70–180 mg/dL, time below 70 mg/dL, time below 54 mg/dL, time above 180 mg/dL, and time above 250 mg/dL were calculated for each age-group and glucose target range. The number of users who selected each target range and the total time spent in each target range were calculated. In addition, for each participant, the number of glucose targets used each day was determined and then averaged across all of the users’ days. If limited data were available for a given glucose target range (<20 users), glycemic outcomes were not reported. The Glycemic Risk Index (GRI) and Continuous Glucose Monitoring Index (COGI) were calculated for each target range and each age-group using the methods described by Eviz et al.^11^ Safety events in the form of episodes of severe hypoglycemia or DKA were collected through the post-market surveillance process and relevant data were inspected to determine which glucose target was used prior to or during these events.

Glucose data were extracted from the CamAPS FX service cloud. Statistical analysis was completed using SAS software, version 9.4 (SAS Institute, NC, USA). Data are presented as mean ± standard deviation for normally distributed data or median (interquartile range) for non-normally distributed data.

## Results

### Demographics of utilization of customized glucose targets

**FIG. 1.**
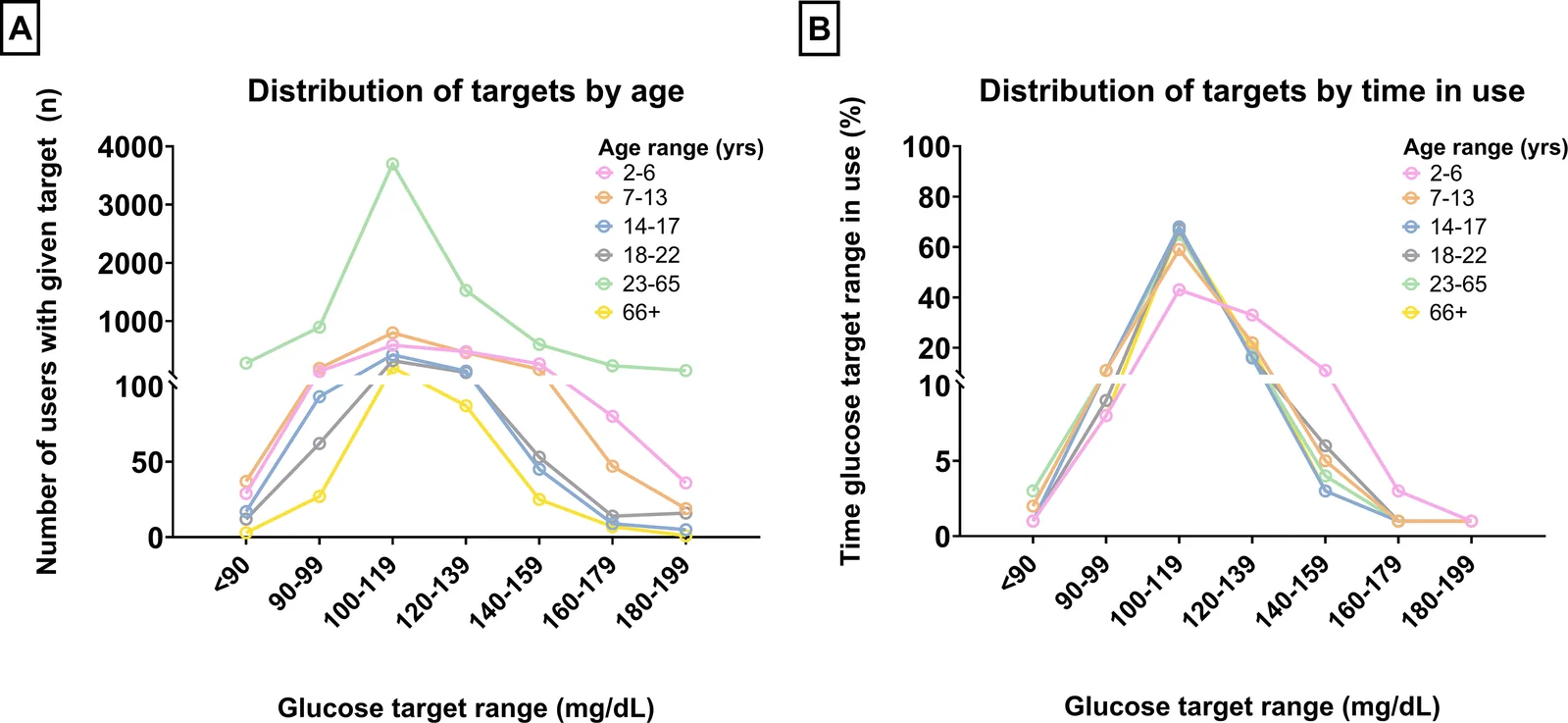
Distribution of personal glucose targets by age (panel A) and time in use (panel B).

A total of 8604 users were included in the analysis, with a mean age of 32 ± 19 years and a median of 89 (IQR 58, 119) days of CGM data available per user. User characteristics and utilized glucose targets are provided in Table 1 and Figure 1. Overall, a wide variety of glucose targets were used across all age-groups. The median of the average number of glucose targets used per day was 1.15 (IQR 1.00, 1.67), with young children and children using slightly more targets per day than older users. The default target was the most frequently used, with 62% of all CGM data coming from the target range that included the default target of 105 mg/dL. After the default target, users most often used a customized target in the range just below or just above the default (10% just below and 19% just above). The extremes of the available targets (<90 mg/dL and >160 mg/dL) were infrequently used, with only 2% of all CGM data coming from the lowest target range, and <1% from the highest.

**Table 1.** User Characteristics and Glycemic Outcomes According to Personal Glucose Target and Age

	Overall (*N* = 8604)	Young children 2–6 years (*N* = 799)	Children 7–13 years (*N* = 1,113)	Adolescents 14–17 years (*N* = 624)	Young adults 18–22 years (*N* = 507)	Adults 23–65 years (*N* = 5268)	Older adults 66+ years (*N* = 293)
Age, years	32 ± 19	5 ± 1	10 ± 2	15 ± 1	20 ± 1	42 ± 11	71 ± 4
Amount of CGM data per user, days	89 (58, 119)	91 (58, 124)	90 (58, 124)	80 (54, 109)	77 (49, 106)	90 (61, 120)	95 (59, 122)
Average number of glucose targets per day, *n*	1.15 (1.00, 1.67)	1.50 (1.00, 2.00)	1.40 (1.00, 1.89)	1.00 (1.00, 1.50)	1.00 (1.00, 1.50)	1.00 (1.00, 1.50)	1.00 (1.00, 1.52)
No of participants using given target, *n*							
<90 mg/dL	378	29	37	17	12	280	3
90–99 mg/dL	1407	138	191	93	62	896	27
100–119 mg/dL	6038	588	801	424	323	3698	204
120–139 mg/dL	2820	479	460	144	121	1529	87
140–159 mg/dL	1167	269	174	45	53	601	25
160–179 mg/dL	392	80	47	9	14	235	7
180–199 mg/dL	230	36	19	5	16	153	1
Amount of CGM data using given target, days (%)							
<90 mg/dL	12,535 (2%)	700 (1%)	1,331 (2%)	446 (1%)	124 (<1%)	9667 (3%)	268 (1%)
90–99 mg/dL	56,802 (10%)	4324 (8%)	8446 (11%)	3702 (11%)	2337 (9%)	36,471 (11%)	1522 (8%)
100–119 mg/dL	345,960 (62%)	24,691 (43%)	44,839 (59%)	23,257 (68%)	16,720 (67%)	223,301 (65%)	13,152 (67%)
120–139 mg/dL	106,233 (19%)	18,781 (33%)	16,523 (22%)	5642 (16%)	4,028 (16%)	57,273 (17%)	3,985 (20%)
140–159 mg/dL	26,169 (5%)	6,181 (11%)	3544 (5%)	977 (3%)	1393 (6%)	13,393 (4%)	681 (3%)
160–179 mg/dL	5310 (<1%)	1459 (3%)	766 (1%)	169 (<1%)	138 (<1%)	2734 (<1%)	45 (<1%)
180–199 mg/dL	3179 (<1%)	691 (1%)	263 (<1%)	61 (<1%)	213 (<1%)	1939 (<1%)	13 (<1%)
Sensor glucose outcomes using given target							
Time 70–180 mg/dL, %							
<90 mg/dL	81 ± 12	74 ± 11	76 ± 10	LD	LD	83 ± 10	LD
90–99 mg/dL	77 ± 12	71 ± 13	74 ± 11	74 ± 14	71 ± 15	79 ± 11	85 ± 9
100–119 mg/dL	72 ± 12	65 ± 13	70 ± 12	71 ± 13	66 ± 14	74 ± 11	78 ± 11
120–139 mg/dL	69 ± 14	64 ± 13	68 ± 14	70 ± 13	64 ± 16	70 ± 13	76 ± 13
140–159 mg/dL	64 ± 16	62 ± 15	63 ± 16	66 ± 15	62 ± 13	65 ± 17	70 ± 16
160–179 mg/dL	60 ± 19	57 ± 15	55 ± 19	LD	LD	62 ± 20	LD
180–199 mg/dL	54 ± 19	55 ± 15	LD	LD	LD	55 ± 19	LD
Time <70 mg/dL, %							
<90 mg/dL	4.5 (2.6, 6.9)	5.0 (2.5, 7.2)	5.4 (3.4, 7.8)	LD	LD	4.2 (2.6, 6.6)	LD
90–99 mg/dL	3.0 (1.8, 4.8)	4.2 (2.9, 6.2)	3.6 (2.4, 5.3)	2.6 (1.6, 4.0)	2.5 (1.6, 3.5)	2.9 (1.6, 4.6)	1.8 (0.6, 2.9)
100–119 mg/dL	2.2 (1.3, 3.6)	3.6 (2.1, 5.3)	2.9 (1.8, 4.3)	2.4 (1.4, 3.7)	1.9 (1.1, 3.1)	2.0 (1.1, 3.2)	1.3 (0.9, 2.3)
120–139 mg/dL	1.7 (0.8, 3.1)	3.0 (1.7, 4.6)	2.4 (1.3, 3.6)	2.1 (1.1, 3.2)	1.5 (0.6, 2.5)	1.3 (0.5, 2.4)	0.8 (0.3, 1.4)
140–159 mg/dL	1.4 (0.4, 3.5)	2.6 (1.1, 4.4)	2.0 (0.8, 3.7)	1.1 (0.3, 2.6)	1.3 (0.3, 2.9)	1.0 (0.3, 2.6)	0.4 (0.0, 1.3)
160–179 mg/dL	0.9 (0.0, 2.4)	2.0 (0.9, 3.5)	1.3 (0.0, 2.9)	LD	LD	0.6 (0.0, 2.2)	LD
180–199 mg/dL	0.8 (0.0, 2.4)	2.0 (0.3, 3.2)	LD	LD	LD	0.5 (0.0, 2.2)	LD
Time <54 mg/dL, %							
<90 mg/dL	0.66 (0.20, 1.23)	0.86 (0.15, 1.53)	0.94 (0.61, 1.77)	LD	LD	0.58 (0.20, 1.13)	LD
90–99 mg/dL	0.45 (0.15, 0.93)	0.75 (0.38, 1.39)	0.66 (0.32, 1.08)	0.41 (0.11, 0.76)	0.33 (0.11, 0.70)	0.39 (0.14, 0.84)	0.09 (0.00, 0.37)
100–119 mg/dL	0.33 (0.13, 0.69)	0.63 (0.29, 1.17)	0.50 (0.22, 0.88)	0.37 (0.17, 0.78)	0.33 (0.14, 0.67)	0.28 (0.11, 0.59)	0.16 (0.07, 0.33)
120–139 mg/dL	0.22 (0.03, 0.58)	0.52 (0.20, 0.98)	0.34 (0.06, 0.71)	0.33 (0.10, 0.54)	0.21 (0.04, 0.46)	0.16 (0.01, 0.43)	0.06 (0.00, 0.22)
140–159 mg/dL	0.13 (0.00, 0.56)	0.40 (0.05, 1.08)	0.24 (0.00, 0.65)	0.14 (0.00, 0.39)	0.17 (0.00, 0.58)	0.07 (0.00, 0.34)	0.02 (0.00, 0.12)
160–179 mg/dL	0.01 (0.00, 0.38)	0.30 (0.00, 0.74)	0.00 (0.00, 0.58)	LD	LD	0.00 (0.00, 0.25)	LD
180–199 mg/dL	0.02 (0.00, 0.39)	0.27 (0.02, 0.74)	LD	LD	LD	0.00 (0.00, 0.28)	LD
Time >180 mg/dL, %							
<90 mg/dL	14 ± 12	21 ± 11	19 ± 11	LD	LD	12 ± 10	LD
90–99 mg/dL	19 ± 12	24 ± 12	22 ± 11	22 ± 14	26 ± 15	17 ± 11	13 ± 8
100–119 mg/dL	25 ± 13	30 ± 13	27 ± 12	26 ± 13	31 ± 14	24 ± 12	21 ± 12
120–139 mg/dL	29 ± 14	33 ± 13	29 ± 14	27 ± 13	34 ± 17	28 ± 14	23 ± 13
140–159 mg/dL	34 ± 17	35 ± 16	34 ± 16	33 ± 16	36 ± 15	33 ± 17	29 ± 16
160–179 mg/dL	38 ± 19	40 ± 16	43 ± 20	LD	LD	37 ± 20	LD
180–199 mg/dL	44 ± 20	43 ± 16	LD	LD	LD	43 ± 20	LD
Time >250 mg/dL, %							
<90 mg/dL	1 (0, 4)	4 (1, 6)	4 (1, 6)	LD	LD	1 (0, 3)	LD
90–99 mg/dL	3 (1, 7)	5 (2, 8)	5 (2, 8)	4 (1, 8)	6 (1, 13)	2 (1, 5)	1 (0, 3)
100–119 mg/dL	5 (2, 10)	8 (4, 13)	7 (4, 11)	6 (3, 11)	9 (4, 16)	4 (2, 8)	3 (1, 5)
120–139 mg/dL	6 (3, 12)	9 (5, 14)	8 (4, 12)	6 (3, 12)	10 (4, 18)	5 (2, 10)	3 (1, 7)
140–159 mg/dL	8 (3, 15)	10 (5, 15)	10 (4, 16)	9 (3, 14)	9 (5, 16)	7 (2, 13)	5 (2, 14)
160–179 mg/dL	10 (4, 18)	12 (7, 20)	14 (8, 21)	LD	LD	8 (2, 15)	LD
180–199 mg/dL	12 (6, 21)	15 (8, 23)	LD	LD	LD	11 (5, 19)	LD
Mean glucose, mg/dL							
<90 mg/dL	130 ± 19	143 ± 15	137 ± 19	LD	LD	127 ± 17	LD
90–99 mg/dL	140 ± 20	146 ± 20	144 ± 18	144 ± 23	152 ± 27	137 ± 19	133 ± 15
100–119 mg/dL	151 ± 21	158 ± 24	153 ± 21	153 ± 24	161 ± 25	149 ± 19	145 ± 17
120–139 mg/dL	158 ± 22	161 ± 21	158 ± 22	155 ± 22	167 ± 29	156 ± 21	151 ± 19
140–159 mg/dL	164 ± 27	165 ± 27	166 ± 27	165 ± 26	169 ± 27	163 ± 27	160 ± 22
160–179 mg/dL	171 ± 29	173 ± 28	179 ± 33	LD	LD	168 ± 28	LD
180–199 mg/dL	179 ± 34	179 ± 30	LD	LD	LD	177 ± 32	LD
GRI							
<90 mg/dL	33 ± 17	43 ± 17	40 ± 14	LD	LD	30 ± 15	LD
90–99 mg/dL	36 ± 16	46 ± 17	41 ± 14	39 ± 17	42 ± 16	33 ± 15	24 ± 12
100–119 mg/dL	42 ± 15	51 ± 15	44 ± 14	42 ± 15	47 ± 15	39 ± 14	34 ± 15
120–139 mg/dL	46 ± 17	53 ± 15	47 ± 17	43 ± 15	50 ± 17	44 ± 17	36 ± 17
140–159 mg/dL	51 ± 19	55 ± 18	52 ± 19	48 ± 17	53 ± 15	50 ± 20	42 ± 21
160–179 mg/dL	56 ± 22	60 ± 17	61 ± 20	LD	LD	54 ± 24	LD
180–199 mg/dL	62 ± 20	61 ± 15	LD	LD	LD	61 ± 21	LD
COGI							
<90 mg/dL	47 ± 5	44 ± 5	45 ± 5	LD	LD	48 ± 4	LD
90–99 mg/dL	45 ± 6	43 ± 6	44 ± 5	44 ± 7	42 ± 7	46 ± 5	48 ± 4
100–119 mg/dL	42 ± 6	40 ± 6	42 ± 6	42 ± 6	40 ± 7	43 ± 6	44 ± 5
120–139 mg/dL	40 ± 7	39 ± 6	40 ± 7	41 ± 6	38 ± 8	41 ± 7	43 ± 6
140–159 mg/dL	38 ± 8	37 ± 8	38 ± 8	39 ± 7	37 ± 7	38 ± 8	40 ± 8
160–179 mg/dL	36 ± 10	35 ± 8	33 ± 10	LD	LD	36 ± 10	LD
180–199 mg/dL	33 ± 10	34 ± 8	LD	LD	LD	33 ± 10	LD

Data are mean ± SD or median (IQR) unless otherwise indicated. LD, limited data available (<20 users).

GRI, Glycemia Risk Index; COGI, Continuous Glucose Monitoring Index.

In terms of utilization of non-default targets by user age, customized targets were used most frequently by very young children (>50% of days), followed by school-aged children (>40%). All other age-groups utilized the default target the majority of the time (65%–68% of the time), with customized targets just above the default being the next most frequently used (120 mg to139 mg/dL). The third most frequently used target was just below the default (90–99 mg/dL) across all age-groups, apart from in very young children, who used the higher target range of 140–159 mg/dL more frequently. For all other age-groups, customized targets higher than 140 mg/dL were infrequently used, and targets lower than 90 mg/dL were rarely used across all age-groups.

### Glycemic outcomes using customized glucose targets

**FIG. 2.**
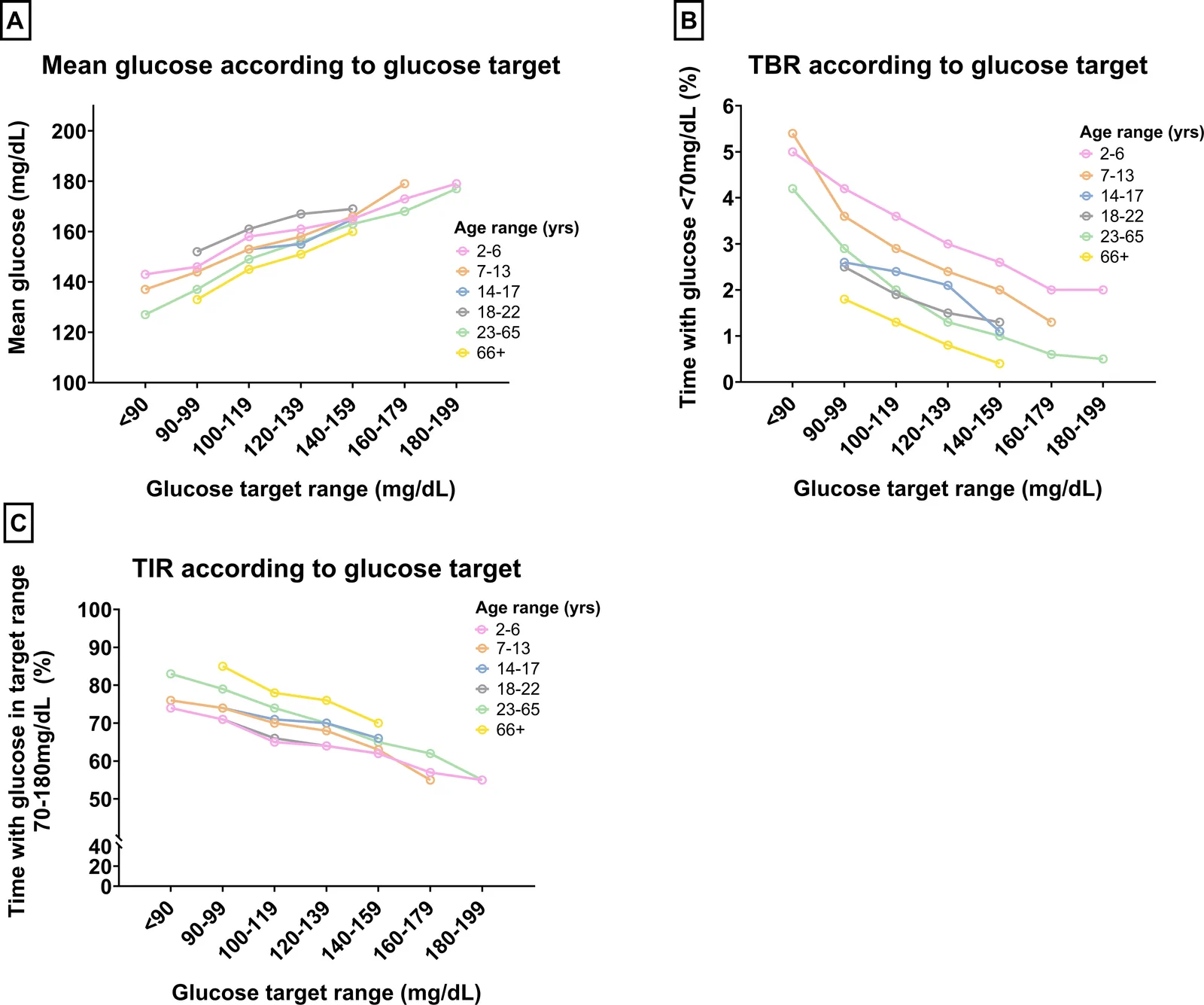
Glycemic outcomes according to personal glucose target and age. Panel A shows mean glucose, panel B time below range (<70 mg/dL), and panel C time in target range (70 to 180 mg/dL) according to glucose target.

Glycemic outcomes using given glucose targets are shown in Table 1 and Figure 2. When analyzing data from all users combined, time in target range 70–180 mg/dL was highest using the lowest glucose targets, dropping as the target increased. Conversely, mean glucose was lowest at the lowest glucose target, rising steadily and linearly with higher target settings. Personal glucose targets higher than 120 mg/dL were associated with time in range less than 70% and time above range >180 mg/dL of more than 25%, thus not being within guideline-recommended limits.^12^ As expected, time below range (<70 mg/dL and <54 mg/dL) was highest at the lowest targets, although remained within guideline-recommended parameters (<4% with glucose <70 mg/dL) apart from at the lowest targets of 80–89 mg/dL (lowest two target settings in young children), with time <54 mg/dL always remaining below the recommended <1%, even at targets 80–89 mg/dL.^12^ Both the GRI and COGI appeared to improve in a linear fashion in line with lower personal glucose target settings.

### Glycemic outcomes using customized glucose targets according to age

On the whole, older adults had the lowest mean glucose, highest time in range, and lowest time below range across all target ranges (Fig. 2). Young adults and very young children tended to have the highest mean glucose and lowest time in range across the different target ranges, generally below the recommended 70%, apart from at the very lowest target settings. All other age-groups tended to have time in range >70% until reaching target range 140–159 mg/dL. The trends observed in time below range appeared age-related across all targets, with very young children having the highest time below range, followed by children and adolescents, and young adults and adults having lower levels of time below range than the pediatric cohort. Reassuringly, all age-groups had <4% time below range as per recommended guidelines, apart from at the lowest possible target setting (lowest two settings in very young children). Percentage time below 54 mg/dL remained <1% across all targets and age-groups.

As a general trend, mean glucose tended to rise with increasingly higher glucose targets across all age-groups. Incremental increases in mean glucose dependent on target range appeared largest when changing from lowest to default target, with flattening of the trajectories evident thereafter. Time in target range followed an inverse trend, with percentages decreasing with every target range increase. In older adults, the decrease in time in range appears sharpest when changing from target range 90–99 mg/dL to the default target, and when changing from 120–139 mg/dL to the higher target range of 140–159 mg/dL. In adults, the changes appear much more linear, whereas in very young children, children, adolescents, and young adults, trajectories appear much flatter. This is with exception of the change from target range 90–99 mg/dL to default target in very young children and the change from target range 140–159 mg/dL to 160–179 mg/dL in children, both of with appeared to be associated with a sharper decline in time in range.

With regard to time below range, lower targets coincided with higher percentage time below range. The largest reductions in time below range were generally observed when moving from the lowest possible to the next lowest and then the default targets, with reductions appearing more linear thereafter. Importantly, we noted little difference in percentage time spent below target range when moving from the second highest target range (160–179 mg/dL) to the highest (180–199 mg/dL) in very young children and adults. When assessing the impact of different glucose targets at nighttime (midnight to 6am), time spent with glucose <70 mg/dL and <54 mg/dL were below recommended targets (<4% and <1%, respectively) at all glucose targets across all age-groups, apart from at the very lowest target in very young children and children (Supplementary Table S1). Notably, nighttime hypoglycemia increased with use of the highest possible target (180–199 mg/dL) compared with the next highest (160–179 mg/dL) in very young children. In a separate analysis, nighttime was expanded for young children to be 8 pm to 6am with the impact of different glucose targets showing similar results with regards to hypoglycemia (Supplementary Table S2).

### Safety outcomes

Over more than 500,000 days of closed-loop use, 11 severe hypoglycemia events, and 13 DKA events were reported (Supplementary Tables S3 and S4). Of the 11 severe hypoglycemia events, four occurred at a target <100 mg/dL (none at target <90 mg/dL), three occurred at or near the default target, two in the target range 100–119 mg/dL, and two at a target >120 mg/dL. Seven of these events appeared to be related to manual overbolusing. With regard to the 13 DKA events, one of these occurred at a target of 99 mg/dL, four occurred at or near the default target, four in the target range of 120–139 mg/dL, two in the range 140–159 mg/dL, and two in the range of 160–179 mg/dL. Closed-loop was not operational during six of these events and four were related to infusion set failures.

## Discussion

The present retrospective analysis of real-world data from >8600 hybrid closed-loop CamAPS FX users highlights the anticipated impact of personal glucose targets on glycemic control, with high variability evident between different age-groups. For the most part, there appeared to be a fairly linear association between higher glucose targets and higher mean glucose, lower time in range, and lower time in hypoglycemia, suggesting that clinical use of personal glucose targets to alter these metrics may well be warranted.

Overall, personal targets above 120 mg/dL were associated with time in target range below the recommended 70%. Their use may therefore only be warranted in particular clinical circumstances and efforts should be made to reduce the glucose target once clinically safe to do so. These results are consistent with an analysis of the impact of applying personal glucose targets using data from several randomized controlled trials with the CamAPS FX algorithm.^10^ Results from real-world analyses of other hybrid closed-loop systems with adjustable glucose targets similarly suggest routine use of the lowest target for optimal outcomes (noting that all other systems have a minimum target of 100 mg/dL).^7,8^ With CamAPS FX, use of the very lowest target (80–89 mg/dL) should perhaps be reserved for specific clinical indications, such as pregnancy, as time in hypoglycemia (<70 mg/dL) was above the recommended 4% in the overall data cohort when using the lowest target, although time <54 mg/dL remained <1%.

In keeping with randomized controlled studies^2,13^ and other real-world data,^14^ older adults had the highest time in range with the lowest burden of hypoglycemia at all glucose targets compared with other age-groups. Time in hypoglycemia (<70 mg/dL) was <2% with time in range of 78% even at targets below 100 mg/dL, suggesting that achieving tight control with the use of lower targets does not come at the expense of frequent hypoglycemia in this vulnerable population.

Very young children and school-aged children had the highest burden of hypoglycemia, in keeping with clinical study and real-world data.^7,8,15–17^ In our analysis, it was notable that targets <100 mg/dL were associated with levels of hypoglycemia above recommended guidelines, suggesting that targets below the default of 105 mg/dL should be used with caution in these age-groups, despite concurrent improvements in time in range. Indeed targets below the default should be used with caution in anyone with high baseline levels of hypoglycemia, as is often the case in very young children.^15^ This is in contrast to adolescents and young adults, whose time below range remained well below the recommended 4% even at lower targets <100 mg/dL, suggesting that targets below the default can safely be used and appear effective in improving time in range. Importantly, a glucose target of >180 mg/dL did not reduce time in hypoglycemia in very young children and adults, while however further reducing time in target range, highlighting that the highest targets may not be beneficial in routine clinical practice unless other considerations apply.

In terms of safety, the number of severe hypoglycemia events was low and occurred across a range of glucose targets, albeit occurring relatively more frequently for those using the lowest glucose targets. However, the majority of the severe hypoglycemia events were associated with manual blousing, suggesting that factors other than the glucose target itself contributed to these events. The low severe hypoglycemia event rate observed in this cohort may be due to the retrospective nature of reporting these events. The number of episodes of DKA was also low and did not appear related to the glucose target setting. Most episodes of DKA were related to either set failures or occurred when closed-loop was not operational.

Strengths of our study include the inclusion of data from a large cohort across a wide range of demographics and from several different countries. The main limitation is the retrospective nature of our analysis. Additional limitations include that glycemic outcomes related to glucose targets represent a snapshot in time; the collection of minimal demographic data (age and type 1 diabetes being the only available variables); as well as the unequal distribution of users across age-groups. Personal glucose targets are user-selectable, thus making selection dependent, at least in part, on personal/parental risk assessment of hypo- and hyperglycemia. We collected no information on users’ motivations for selecting different personal glucose targets.

## Conclusions

In summary, when using the CamAPS FX hybrid closed-loop app, personal glucose targets are frequently utilized across different age-groups and their use is associated with clinical differences in glycemic control. The clinical impact of various targets differs depending on user age. Clinicians should always consider individual factors beyond age, such as glycemic variability, bolusing behavior, exercise habits, etc. when suggesting glucose target adjustments. Our analysis suggests that targets may be modified to alter glucose outcomes and may help health care providers guide users in adjusting the personal glucose target to better achieve recommended glycemic targets as well as individual glycemic goals.

## Authors’ Contributions

S.B., P.C., M.E.W., and R.H. co-designed the analysis. S.B., P.C., M.E.W., Y.R., J.W., and R.H. carried out or supported data analysis, including statistical analysis. J.W. wrote the article. R.H. designed the control algorithm. S.B., M.E.W., J.S., P.C., Y.R., and R.H. critically reviewed the article. S.B., J.W., and R.H. are the guarantors of this work and, as such, had full access to all the data in the study and take responsibility for the integrity of the data and the accuracy of the data analysis.

## Supplemental Material

sj-pdf-1-dtt-10.1177_15209156251376010 — Supplemental material for Real-World Clinical Impact of Using Personal Glucose Targets in a Hybrid Closed-Loop System Differs According to Age Supplemental material, sj-pdf-1-dtt-10.1177_15209156251376010 for Real-World Clinical Impact of Using Personal Glucose Targets in a Hybrid Closed-Loop System Differs According to Age by
