# Supplementary material for: Real-World Clinical Impact of Using Personal Glucose Targets in a Hybrid Closed-Loop System Differs According to Age
Source: Diabetes Technol Ther. 2025 Sep 5;28(2):167–74. doi: 10.1177/15209156251376010 (PMC13535542; doi:10.1177/15209156251376010)
Supplement: Supplementary file 1 [file sj-pdf-1-dtt-10.1177_15209156251376010.pdf]

Supplementary Appendix

This appendix has been provided by the authors to give readers additional information about their work.

Table of Contents:

Figure S1. Automated closed-loop insulin delivery system: CamAPS FX. 2

Table S1. Glycemic outcomes according to personal glucose target and age in the nighttime period (00:00 to 05:59). 3

Table S2. Glycemic outcomes for young children (2 to 6 years) according to personal glucose target in the redefined nighttime period (20:00 to 05:59). 7

Table S3. Details of severe hypoglycemia events during real-world closed-loop use. 8

Table S4. Details of diabetic ketoacidosis (DKA) events during real-world closed-loop use. . 9

Figure S1. Automated closed-loop insulin delivery system: CamAPS FX.

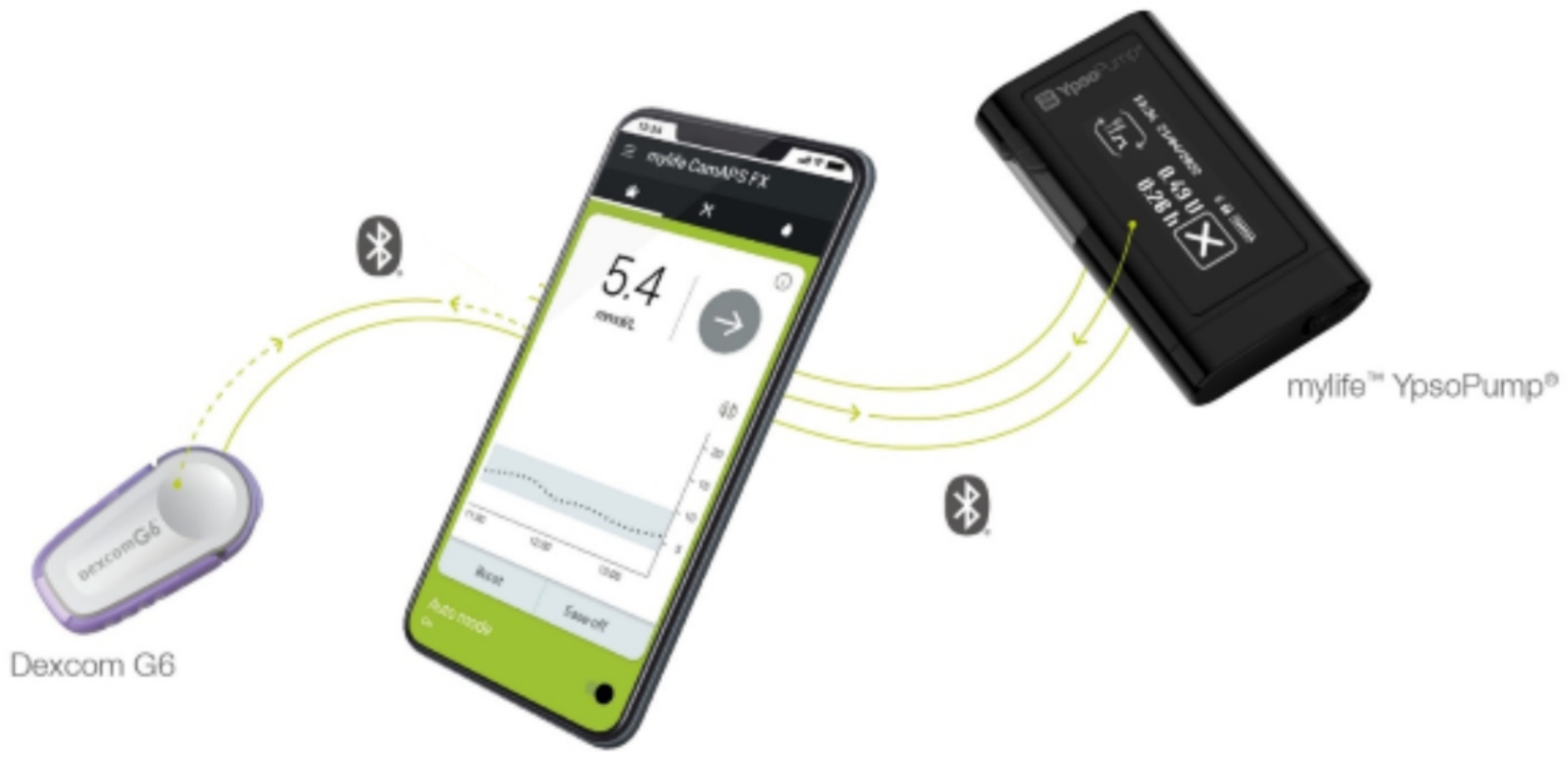

CamAPS FX comprises (i) unlocked smartphone hosting (ii) the CamAPS FX app (CamDiab, UK) running the Cambridge model predictive control algorithm (version 0.3.71), (iii) YpsoPump insulin pump (Ypsomed AG, Switzerland), and (iv) Dexcom G6 continuous glucose monitor (Dexcom, CA, USA). Both pump and sensor transmitter communicate wirelessly over Bluetooth with the CamAPS FX app hosted on the phone. The control algorithm is initialized using total daily insulin dose and body weight, and incorporates adaptive learning with regards to overall, diurnal and prandial insulin requirements. The treat-to-target algorithm has a nominal glucose target of 105mg/dL, which is user-adjustable between 80 and 198mg/dL across different times of day. The CamAPS FX app streams data to Glooko data ecosystem (Glooko, Sweden) for remote data visualization and provides real-time SMS alerts to parents/guardians. CamAPS FX can be configured to operate in mirror mode, enabling one user's app to display real-time data from another CamAPS FX user—suitable for monitoring by parents, guardians, or caregivers. The app containes a bolus calculator to initiate bolus delivery from the phone, a user-selectable 'Ease-off' mode to reduce insulin delivery around activity/exercise periods, and a 'Boost' mode to intensify insulin delivery when required.

Table S1. Glycemic outcomes according to personal glucose target and age in the nighttime period (00:00 to 05:59).

	**Overall (N=8,604)**	**Young children 2-6 years (N=799)**	**Children 7-13 years (N=1,113)**	**Adolescents 14-17 years (N=624)**	**Young adults 18-22 years (N=507)**	**Adults 23-65 years (N=5,268)**	**Older adults 66+ years (N=293)**
**Age, years**	32 ± 19	5 ± 1	10 ± 2	15 ± 1	20 ± 1	42 ± 11	71 ± 4
**Amount of CGM data per user, days**	22 (15, 30)	23 (15, 31)	23 (15, 31)	20 (14, 28)	20 (13, 27)	23 (15, 30)	24 (15, 31)
**No of participants using given target, n**							
<90mg/dL	332	25	33	16	6	250	2
90-99mg/dL	1,294	124	178	82	58	829	23
100-119mg/dL	5,818	544	760	408	314	3,594	198
120-139mg/dL	2,495	431	375	133	110	1,360	86
140-159mg/dL	912	213	120	38	47	477	17
160-179mg/dL	265	67	28	7	11	147	5
180-199mg/dL	153	27	13	4	14	94	1
**Amount of CGM data using given target, days (%)**							
<90mg/dL	3,197 (2%)	181 (1%)	341 (2%)	113 (1%)	32 (<1%)	2,461 (3%)	68 (1%)
90-99mg/dL	14,388 (10%)	1,095 (8%)	2,155 (11%)	945 (11%)	591 (9%)	9,219 (11%)	382 (8%)
100-119mg/dL	87,318 (62%)	6,215 (43%)	11,404 (59%)	5,918 (68%)	4,236 (67%)	56,235 (65%)	3,310 (67%)
120-139mg/dL	26,786 (19%)	4,740 (33%)	4,179 (22%)	1,441 (17%)	1,018 (16%)	14,403 (17%)	1,005 (20%)
140-159mg/dL	6,536 (5%)	1,555 (11%)	882 (5%)	249 (3%)	350 (6%)	3,334 (4%)	165 (3%)
160-179mg/dL	1,300 (<1%)	369 (3%)	188 (<1%)	42 (<1%)	34 (<1%)	657 (<1%)	9 (<1%)
180-199mg/dL	775 (<1%)	173 (1%)	65 (<1%)	14 (<1%)	55 (<1%)	464 (<1%)	3 (<1%)
**Sensor glucose outcomes using given target**							
**Time 70-180mg/dL, %**							
<90mg/dL	84 ± 12	75 ± 12	82 ± 10	LD	LD	85 ± 11	LD

90-99mg/dL	82 ± 12	78 ± 13	80 ± 12	83 ± 13	78 ± 15	83 ± 12	91 ± 8
100-119mg/dL	78 ± 13	72 ± 14	78 ± 12	78 ± 12	71 ± 15	79 ± 13	85 ± 11
120-139mg/dL	74 ± 15	71 ± 13	75 ± 15	78 ± 14	68 ± 16	75 ± 15	81 ± 15
140-159mg/dL	69 ± 18	68 ± 17	71 ± 16	74 ± 17	66 ± 17	69 ± 19	LD
160-179mg/dL	65 ± 21	64 ± 17	69 ± 20	LD	LD	66 ± 23	LD
180-199mg/dL	58 ± 23	63 ± 14	LD	LD	LD	58 ± 25	LD
**Time <70mg/dL, %**							
<90mg/dL	3.6 (2.0, 6.2)	4.7 (3.4, 9.2)	4.7 (2.4, 7.8)	LD	LD	3.4 (1.9, 5.7)	LD
90-99mg/dL	2.3 (1.2, 4.1)	3.2 (1.7, 5.7)	2.3 (1.2, 3.7)	1.8 (0.9, 3.2)	2.3 (1.2, 4.3)	2.2 (1.1, 4.1)	1.4 (0.5, 2.2)
100-119mg/dL	1.7 (0.8, 3.0)	3.0 (1.4, 5.2)	1.7 (0.9, 3.0)	1.7 (1.0, 3.0)	2.0 (0.7, 3.3)	1.5 (0.7, 2.8)	0.9 (0.3, 1.8)
120-139mg/dL	1.1 (0.3, 2.4)	2.3 (1.0, 4.3)	1.2 (0.3, 2.4)	1.2 (0.3, 2.1)	1.0 (0.3, 1.7)	0.9 (0.2, 2.1)	0.3 (0.0, 0.9)
140-159mg/dL	0.8 (0.1, 2.4)	1.7 (0.5, 4.0)	0.7 (0.1, 2.2)	0.5 (0.0, 2.5)	0.8 (0.1, 2.1)	0.5 (0.0, 1.8)	LD
160-179mg/dL	0.5 (0.0, 2.0)	1.3 (0.4, 3.2)	0.4 (0.0, 1.0)	LD	LD	0.4 (0.0, 1.8)	LD
180-199mg/dL	0.4 (0.0, 2.2)	2.2 (0.3, 3.7)	LD	LD	LD	0.2 (0.0, 2.0)	LD
**Time <54mg/dL, %**							
<90mg/dL	0.47 (0.00, 1.12)	1.12 (0.31, 2.53)	0.71 (0.23, 1.42)	LD	LD	0.40 (0.00, 0.90)	LD
90-99mg/dL	0.32 (0.03, 0.82)	0.46 (0.04, 1.08)	0.30 (0.05, 0.85)	0.30 (0.00, 0.69)	0.39 (0.09, 0.84)	0.31 (0.03, 0.80)	0.10 (0.00, 0.38)
100-119mg/dL	0.24 (0.05, 0.63)	0.46 (0.12, 1.19)	0.24 (0.06, 0.62)	0.29 (0.08, 0.70)	0.39 (0.06, 0.75)	0.23 (0.04, 0.57)	0.08 (0.00, 0.24)
120-139mg/dL	0.12 (0.00, 0.48)	0.33 (0.06, 0.91)	0.13 (0.00, 0.46)	0.16 (0.00, 0.45)	0.14 (0.00, 0.42)	0.08 (0.00, 0.39)	0.02 (0.00, 0.12)
140-159mg/dL	0.04 (0.00, 0.43)	0.16 (0.00, 0.84)	0.07 (0.00, 0.28)	0.01 (0.00, 0.22)	0.10 (0.00, 0.38)	0.00 (0.00, 0.32)	LD
160-179mg/dL	0.00 (0.00, 0.32)	0.16 (0.00, 0.84)	0.00 (0.00, 0.21)	LD	LD	0.00 (0.00, 0.25)	LD
180-199mg/dL	0.00 (0.00, 0.47)	0.63 (0.00, 0.93)	LD	LD	LD	0.00 (0.00, 0.23)	LD
**Time >180mg/dL, %**							
<90mg/dL	11 ± 11	19 ± 10	13 ± 10	LD	LD	10 ± 10	LD
90-99mg/dL	15 ± 12	18 ± 12	17 ± 12	15 ± 13	19 ± 15	14 ± 11	7 ± 8
100-119mg/dL	20 ± 13	24 ± 14	20 ± 12	19 ± 12	26 ± 16	19 ± 13	13 ± 11
120-139mg/dL	24 ± 15	26 ± 14	23 ± 15	21 ± 14	30 ± 16	24 ± 15	18 ± 15

140-159mg/dL	29 ± 18	29 ± 17	27 ± 16	25 ± 17	32 ± 16	29 ± 20	LD
160-179mg/dL	33 ± 21	33 ± 17	31 ± 20	LD	LD	33 ± 23	LD
180-199mg/dL	40 ± 24	34 ± 14	LD	LD	LD	40 ± 26	LD
**Time >250mg/dL, %**							
<90mg/dL	1 (0, 3)	2 (1, 6)	1 (0, 5)	LD	LD	0 (0, 2)	LD
90-99mg/dL	2 (0, 4)	2 (0, 5)	2 (1, 5)	2 (1, 5)	3 (1, 7)	1 (0, 3)	0 (0, 1)
100-119mg/dL	3 (1, 7)	4 (1, 8)	3 (1, 7)	3 (1, 8)	6 (2, 12)	3 (1, 6)	1 (0, 3)
120-139mg/dL	4 (1, 8)	5 (2, 8)	4 (1, 8)	3 (1, 7)	8 (3, 15)	4 (1, 7)	1 (0, 4)
140-159mg/dL	5 (1, 10)	5 (2, 10)	4 (1, 9)	5 (0, 8)	5 (1, 14)	4 (1, 11)	LD
160-179mg/dL	5 (1, 12)	5 (2, 10)	5 (2, 11)	LD	LD	5 (0, 13)	LD
180-199mg/dL	9 (3, 18)	9 (4, 13)	LD	LD	LD	8 (2, 19)	LD
**Mean glucose, mg/dL**							
<90mg/dL	125 ± 20	138 ± 16	129 ± 17	LD	LD	123 ± 18	LD
90-99mg/dL	134 ± 19	138 ± 19	138 ± 18	133 ± 18	140 ± 25	132 ± 19	123 ± 16
100-119mg/dL	144 ± 20	149 ± 20	145 ± 18	143 ± 20	154 ± 26	143 ± 20	136 ± 17
120-139mg/dL	151 ± 21	152 ± 20	151 ± 21	148 ± 22	162 ± 25	151 ± 22	146 ± 19
140-159mg/dL	159 ± 27	158 ± 26	157 ± 25	158 ± 32	165 ± 28	159 ± 29	LD
160-179mg/dL	164 ± 30	162 ± 25	162 ± 27	LD	LD	165 ± 33	LD
180-199mg/dL	173 ± 38	162 ± 21	LD	LD	LD	173 ± 39	LD
**GRI**							
<90mg/dL	28 ± 18	43 ± 19	31 ± 15	LD	LD	26 ± 17	LD
90-99mg/dL	29 ± 17	37 ± 20	31 ± 16	27 ± 16	33 ± 19	27 ± 17	15 ± 12
100-119mg/dL	34 ± 18	44 ± 19	34 ± 17	32 ± 16	42 ± 19	33 ± 17	23 ± 15
120-139mg/dL	38 ± 20	45 ± 18	36 ± 20	33 ± 19	44 ± 20	37 ± 19	29 ± 20
140-159mg/dL	45 ± 23	48 ± 21	42 ± 21	37 ± 20	48 ± 21	44 ± 24	LD
160-179mg/dL	50 ± 27	54 ± 23	45 ± 26	LD	LD	48 ± 28	LD
180-199mg/dL	57 ± 27	55 ± 19	LD	LD	LD	57 ± 30	LD

**COGI**							
<90mg/dL	48 ± 5	45 ± 5	48 ± 4	LD	LD	49 ± 4	LD
90-99mg/dL	47 ± 5	45 ± 6	46 ± 6	47 ± 6	45 ± 7	47 ± 5	50 ± 3
100-119mg/dL	45 ± 6	42 ± 6	45 ± 6	45 ± 5	42 ± 7	45 ± 6	47 ± 5
120-139mg/dL	42 ± 7	42 ± 6	43 ± 7	44 ± 7	40 ± 7	43 ± 7	45 ± 7
140-159mg/dL	40 ± 9	40 ± 8	41 ± 8	42 ± 8	38 ± 8	40 ± 10	LD
160-179mg/dL	38 ± 10	38 ± 8	39 ± 10	LD	LD	38 ± 11	LD
180-199mg/dL	34 ± 12	38 ± 7	LD	LD	LD	34 ± 13	LD

Data are mean ± SD or median (IQR) unless otherwise indicated. LD – limited data available (<20 users).

Table S2. Glycemic outcomes for young children (2 to 6 years) according to personal glucose target in the redefined nighttime period (20:00 to 05:59).

Glucose Target	Time 70-180mg/dL	Time <70mg/dL	Time <54mg/dL	Time >180mg/dL	Time >250mg/dL	Mean glucose	GRI	COGI
<90mg/dL	70 ± 13	4.8 (2.8, 9.0)	0.97 (0.23, 2.16)	25 ± 13	5 (1, 8)	147 ± 19	49 ± 19	42 ± 6
90-99mg/dL	72 ± 14	3.8 (2.3, 6.1)	0.63 (0.23, 1.28)	23 ± 13	4 (2, 8)	145 ± 20	44 ± 19	43 ± 6
100-119mg/dL	66 ± 13	3.2 (1.7, 5.2)	0.58 (0.20, 1.21)	30 ± 13	7 (3, 12)	157 ± 21	51 ± 17	40 ± 6
120-139mg/dL	65 ± 13	2.8 (1.3, 4.3)	0.46 (0.16, 0.97)	31 ± 13	8 (4, 13)	160 ± 19	51 ± 16	39 ± 6
140-159mg/dL	62 ± 16	2.1 (0.7, 4.1)	0.31 (0.00, 1.00)	35 ± 16	9 (4, 14)	166 ± 26	54 ± 19	37 ± 8
160-179mg/dL	58 ± 15	1.7 (0.9, 3.6)	0.22 (0.00, 0.87)	38 ± 16	10 (5, 17)	170 ± 26	60 ± 20	36 ± 7
180-199mg/dL	57 ± 14	1.8 (0.7, 3.1)	0.48 (0.21, 0.75)	41 ± 14	14 (6, 20)	173 ± 23	60 ± 17	35 ± 7

Table S3. Details of severe hypoglycemia events during real-world closed-loop use.

Time of day	Glucose target (mg/dL)	Details
Afternoon	90	Series of correction boluses while using Ease-off
Evening	95	Overcorrection based on an incorrect fingerprick glucose
Overnight	99	Severe hypoglycemia due to miscarriage/stillbirth
Morning	99	Related to breakfast bolus
Overnight	104	Incorrect initialization
Morning	104	Unexplained drop in glucose levels
Morning	108	Sensor over-read
Morning	117	2 correction boluses delivered via pump
Overnight	117	Boost followed by overcorrection bolus
Evening	126	Accidental overbolus
Evening	144	2 correction boluses delivered via pump

Table S4. Details of diabetic ketoacidosis (DKA) events during real-world closed-loop use.

Time of day	Glucose target (mg/dL)	Details
Evening	99	Disconnection from closed-loop app. Possibly pump battery removed.
Evening	99-108	Boost on most of the day – likely infusion set failure.
Morning	104	Alcohol intake and vomiting. Phone not near user overnight. System reverted to open loop and lost CGM connection due to distance so no alerts.
Unknown	104	Infusion set failure. No response to high alerts.
Unknown	104	On pre-set pump basal only (closed-loop not operational) and no bolus insulin given.
Morning	121	Pump not put back in run mode following set change, i.e. suspended. Numerous alerts not acted on.
Unknown	121	Infusion set failure.
Unknown	121	Infusion set failure.
Unknown	126	Sensor under-reading.
Unknown	140	Hospitalized for an unrelated illness and closed-loop not operational at the time.
Evening	144	Infusion set failure.
Morning	162	CGM signal loss, closed-loop not operational. Sub-optimal pre-set basal rates.
Afternoon	162	Unrelated concurrent illness and closed-loop restarted while acutely unwell.
